# Research on the Threshold Determination Method of the Duffing Chaotic System Based on Improved Permutation Entropy and Poincaré Mapping

**DOI:** 10.3390/e25121654

**Published:** 2023-12-13

**Authors:** Jing Zhou, Yaan Li, Mingzhou Wang

**Affiliations:** 1 School of Marine Science and Technology, Northwestern Polytechnical University, Xi’an 710072, China; zhoujing705@mail.nwpu.edu.cn; 2 Xi’an Precision Machinery Research Institute, Science and Technology on Underwater Information and Control Laboratory, Xi’an 710077, China

**Keywords:** Duffing system, improved permutation entropy, Poincaré mapping, weak signal detection

## Abstract

The transition from a chaotic to a periodic state in the Duffing chaotic oscillator detection system is crucial in detecting weak signals. However, accurately determining the critical threshold for this transition remains a challenging problem. Traditional methods such as Melnikov theory, the Poincaré section quantitative discrimination method, and experimental analyses based on phase diagram segmentation have limitations in accuracy and efficiency. In addition, they require large computational data and complex algorithms while having slow convergence. Improved permutation entropy incorporates signal amplitude information on the basis of permutation entropy and has better noise resistance. According to the characteristics of improved permutation entropy, a threshold determination method for the Duffing chaotic oscillator detection system based on improved permutation entropy (IPE) and Poincaré mapping (PM) is proposed. This new metric is called Poincaré mapping improved permutation entropy (PMIPE). The simulation results and the verification results of real underwater acoustic signals indicate that our proposed method outperforms traditional methods in terms of accuracy, simplicity, and stability.

## 1. Introduction

In recent years, the study of signal detection methods based on the Duffing chaotic oscillator has gained significant attention in the field of weak signal detection [1,2,3,4]. This approach leverages the sensitivity of the chaotic oscillator to extremely weak periodic signals and its immunity to noise. The Duffing oscillator detection system can be expressed as $d^{2}x(t)/dt^{2}+\mu \cdot dx(t)/dt+ax+bx^{3}=r\cos(\omega t)$, where μ, a, b, r are the parameters that can influence the characteristics of the system. Prior to utilizing the Duffing system for target signal detection, a driving force r with the same frequency as the target signal is preset in the system, and the amplitude of the driving force is adjusted to bring the system to the critical chaotic state. Let s(t) be the signal to be detected, when s(t) is input into the Duffing system, the equation transforms to $d^{2}x(t)/dt^{2}+\mu \cdot dx(t)/dt+ax+bx^{3}=r\cos(\omega t)+s(t)$. The presence of the target signal is established by examining the system’s state before and after the signal is introduced. If the system remains in a chaotic state, it signifies that the signal under test lacks a target signal. On the other hand, if the system transitions to a periodic state, it indicates the existence of the target signal [5]. The size of the driving force added to the Duffing chaotic oscillator system directly determines whether the system is in a chaotic or periodic state. Thus, the threshold of the driving force in the Duffing chaotic oscillator is a crucial parameter that significantly impacts the efficiency of detecting weak signals, and its solution is an essential prerequisite for chaos oscillator detection [6,7,8,9]. However, as what will be discussed in Section 2, this threshold that leads the system to the critical chaotic state is varied owing to the influence of noise and the frequency to be detected. Hence, it is of great importance to determine this parameter quickly and accurately. It is noteworthy that the issue of determining the threshold for the Duffing system is not equivalent to the problem of chaos identification. Near the threshold, the system undergoes a transition from a critical chaotic state to a periodic state and remains invariant thereafter. Therefore, the key to confirming the threshold lies in identifying when the system state undergoes a transition and remains stable.

Several scholars have investigated threshold determination methods, including Melnikov analysis [10], phase diagram method [11], power spectrum method [12], Poincaré section method [13,14,15], and 0–1 test [16]. However, these methods have their limitations. The Melnikov theory analysis method has a low solution accuracy and a high implementation difficulty, while the phase diagram method has a poor computational accuracy and lacks adaptability. The power spectrum analysis method is unable to distinguish between periodic, random noise and chaotic signals effectively, and the Poincaré section method is subjective and not suitable for automatic recognition by computers. According to [17], the weakness of the abovementioned methods are listed in Table 1.

Entropy is commonly used to measure the complexity of a signal, with higher entropy values indicating greater chaos [18,19]. There are several popular entropy algorithms, including approximate entropy [20,21], sample entropy [22], and permutation entropy [23,24]. However, these algorithms have limitations, such as the high computational complexity of the sample entropy algorithm and the low signal resolution of the permutation entropy algorithm [25,26,27]. To address these issues, Chen et al. proposed an Improved Permutation Entropy (IPE) algorithm in 2019, which considers both the amplitude and order information of signals and has good anti-noise performance [28].

In 2020, Huang Ze-hui proposed a threshold determination method based on multi-scale entropy to overcome the limitations of the Duffing system’s current method [29]. This approach leverages the differences in the multi-scale entropy of the Duffing system in different states. However, this method requires a repeated calculation of the sequence’s entropy value to find the most complex sub-sequence and its corresponding multiscale entropy value, resulting in significant computational complexity and its not being suitable for the real-time detection of the system.

This paper proposes a method for determining the threshold and state of a Duffing chaotic oscillator detection system based on Improved Permutation Entropy and Poincaré section theory using the PMIPE approach. The goal is to address the problem of threshold determination in Duffing systems. This method involves adding different driving forces to a Duffing oscillator system with predetermined frequencies and parameters in a weak signal detection system, using the IPE algorithm to calculate the complexity of the Poincaré section sequence under different driving forces, and comparing entropy values to determine whether the system is in a periodic or chaotic state This process enables the determination of the threshold of the Duffing chaotic oscillator detection system. Compared to the method based on multi-scale entropy, this approach is simpler, more efficient, and suitable for real-time signal detection. Unlike the multi-scale entropy method, it does not require the calculation of the entropy value for the entire sequence or the determination of the maximum entropy value of the system.

## 2. Duffing Chaotic Oscillator Detection System

### 2.1. Duffing Oscillator System

The Duffing oscillator, a widely studied chaotic oscillator renowned for its intricate dynamics, is defined by a nonlinear term in its equation. Initially derived from the nonlinear dynamical equation describing the forced oscillations of a damped pendulum, the Duffing oscillator model is a result of considering a damped and periodically driven elastic system with high-order powers disregarded. The equation of motion for the undamped and undriven elastic system is given as:

(1) $$d^{2}x/dt^{2}+ax+bx^{3}=0$$

After the inclusion of damping and periodic driving forces, the Duffing equation is obtained:

(2) $$d^{2}x/dt^{2}+\mu \cdot dx/dt+ax+bx^{3}=r\cos(\omega t)$$

Here, μ represents the damping ratio, and $ax+bx^{3}$ denotes the nonlinear term, with a and b being positive real-number system parameters. The amplitude of the driving force is denoted by r, while ω refers to the circular frequency of the periodic driving force. In this study, μ=0.5, a=1, b=1 were used, respectively. Moreover, all experiments in this study were conducted on a computer with an Intel(R) Core(TM) i7-10510U CPU.

By adjusting the amplitude of the driving force, the Duffing system traverses a range of states, including the initial state, homo-clinic orbit state, bifurcation state, chaotic state, and periodic state. Equation (2) is a simple deterministic equation with a unique solution x(t), but the presence of the nonlinear term in the equation gives rise to complex dynamical properties, such as chaotic behavior. Numerical methods, such as the fourth-order Runge–Kutta algorithm, are required to simulate and solve the nonlinear differential equation and obtain a solution.

### 2.2. Impact of Noise on Duffing Oscillator System

In weak signal detection, target signals are often accompanied by noise, which can affect the system itself. By adding a noise signal to Equation (2), the Duffing equation can be written as follows:

(3) $$d^{2}x(t)/dt^{2}+\mu \cdot dx(t)/dt+ax+bx^{3}=r\cos(\omega t)+\Delta n\left(t\right),$$

where $\Delta n\left(t\right)$ denotes the noise signal with a variance of Δ.

**Figure 1:**
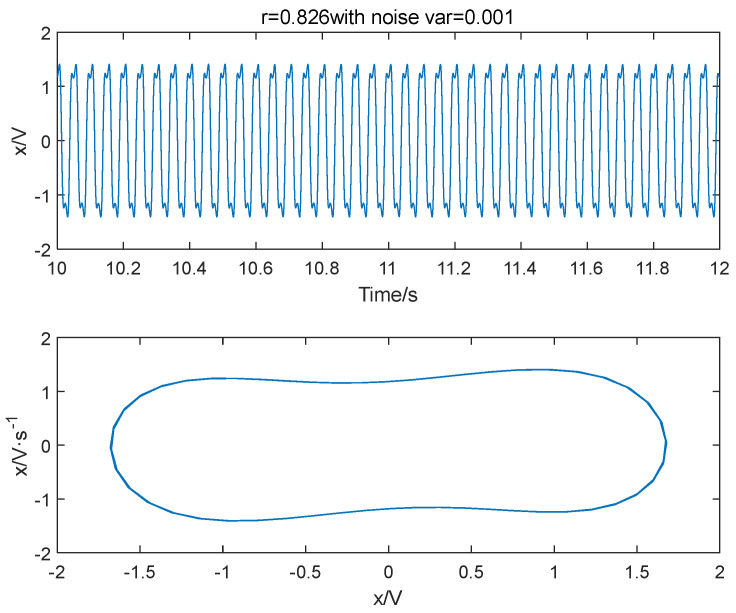
The system is in a periodic state when the variance of noise signal is 0.001 and the power amplitude r = 0.826.

**Figure 2:**
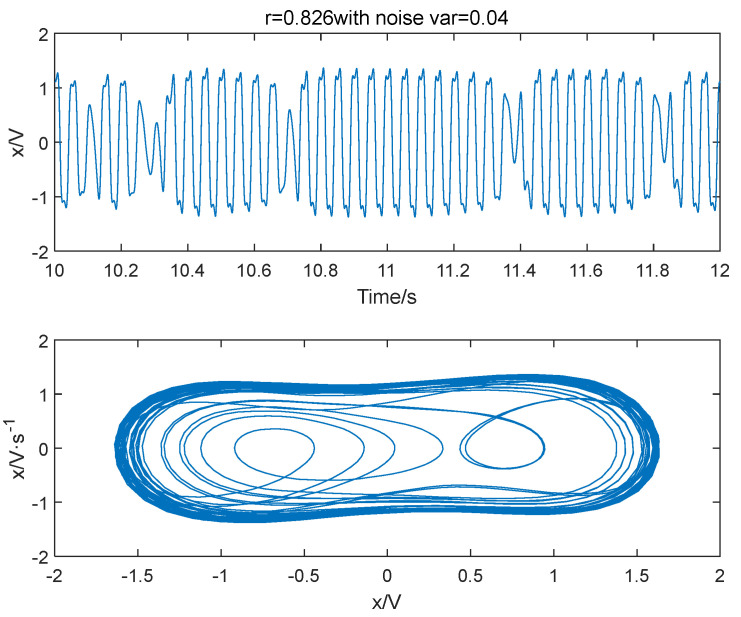
The system is in a chaotic state when the variance of noise signal is 0.04 and the power amplitude r = 0.826.

**Figure 3:**
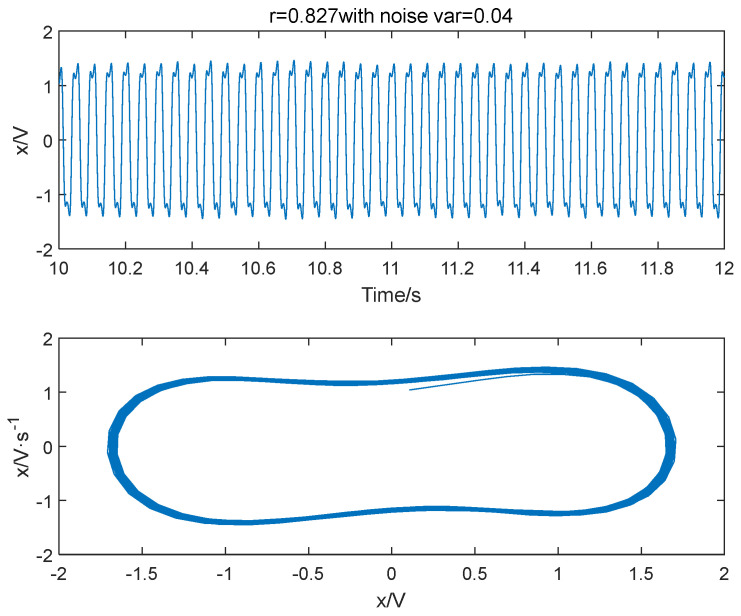
The system is in a periodic state when the variance of noise signal is 0.04 and the power amplitude r = 0.827.

Different variance noise signals are added to the Duffing oscillator system in Equation (3); let ω=2π×20, the system outcomes are shown in Figure 1, Figure 2 and Figure 3. It is seen that the variance of noise directly influences the dynamics of outcome of the system. When r = 0.826 and Δ=0.001, the system is in a periodic state. As Δ increases to 0.04, the system is in a chaotic state. The system will go back to a periodic state if the driving force is improved to 0.827. Therefore, the system requires a larger driving force value to enter the periodic state when there is more noise. As noise increases, the degree of disorder in the system increases. To transition from disorder to order, the ordered components within the system must be strengthened to overcome the increasing disorder. Furthermore, the enhancement of external noise leads to an increase in the chaotic critical threshold.

The results above indicate a slight impact of noise on the threshold, with a marginal increase observed as the noise level rises. It is important to note that considering noise in threshold calculations is applicable only when the noise is stationary and its characteristics are known. This involves calculating the threshold using Equation (3). However, if the noise is non-stationary or its characteristics are unknown, it is advisable to disregard the influence of noise in threshold calculations, and the threshold can be determined using Equation (2).

## 3. Poincaré Mapping Improved Permutation Entropy

### 3.1. Poincaré Mapping

The Poincaré section is a geometric method proposed by the renowned physicist Poincaré in the late 19th century. It selects a suitable section in the multi-dimensional phase space and analyzes the properties of nonlinear systems by observing the distribution of intersection points between the section and the system trajectory. This method replaces the N-order continuous system flow with an N-1 order discrete system and reduces the system order while ensuring that the limit set of the discrete system corresponds to the limit set of the continuous system flow.

In the n-dimensional phase space ($x_{1}$, $dx_{1}/dt$, $x_{2}$, $dx_{2}/dt$, ⋯, $x_{n}$, $dx_{n}/dt$), a section is chosen appropriately, and a Poincaré section is defined by fixing a pair of conjugate variables ($x_{i}$, $dx_{i}/dt$) at a certain value on this section. As the Poincaré section intersects with the system trajectory, it maps the continuous trajectory in the original phase space to a series of discrete points on the section, represented as $P_{n+1}=TP_{n}$ (with T being the Poincaré map). The Poincaré section exhibits the following patterns:

(1) When there is a fixed point or a few discrete points on the Poincaré section, the motion trajectory is periodic;
(2) When the Poincaré section consists of dense points with self-similar structures, the motion trajectory is chaotic.

**Figure 4:**
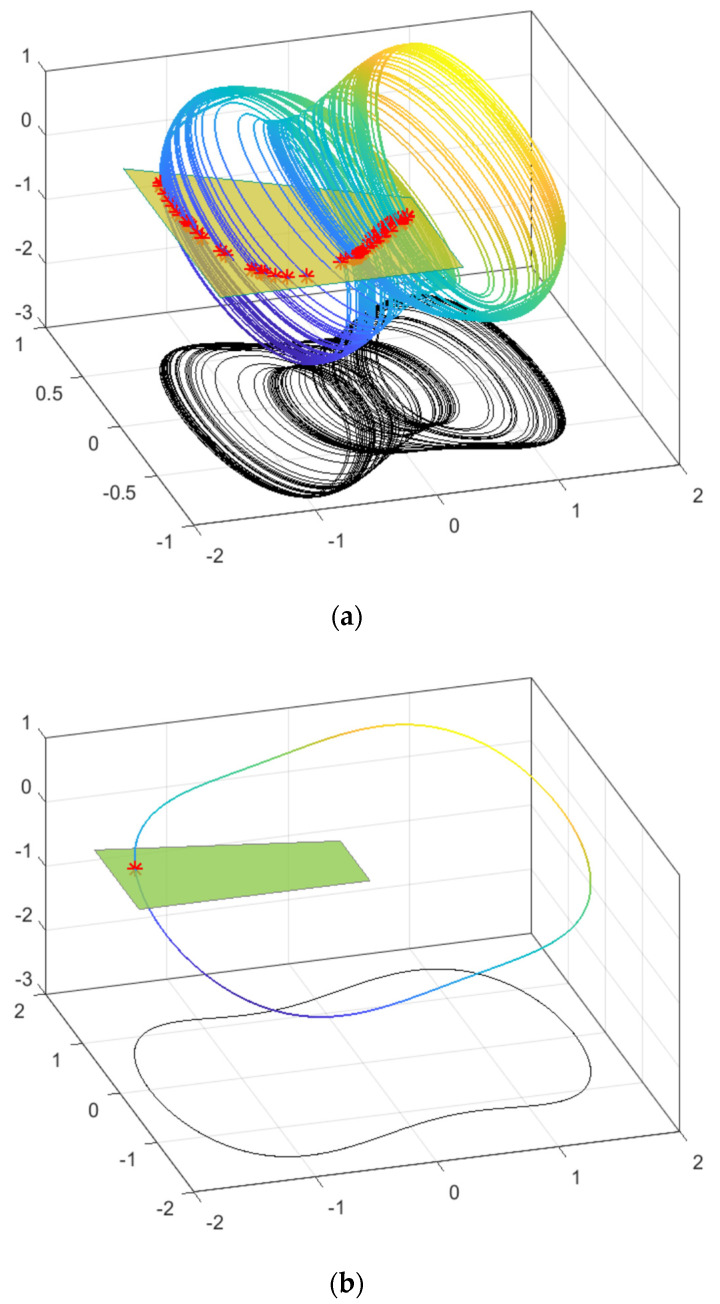
Intersection diagram of Duffing system and Poincaré section in different states. The * represent the intersection point of the Duffing system and Poincaré cross-section; The colored lines represent the phase trajectory of a duffing system. (**a**) Three-dimensional diagram of the intersection of chaotic states and Poincaré sections of Duffing system; (**b**) three-dimensional diagram of the intersection of periodic states and Poincaré sections of Duffing system.

Figure 4 illustrates the intersection points between the Duffing system and the Poincaré section in different states. The three-dimensional and two-dimensional plots of the intersection points in the chaotic state of the Duffing system and the Poincaré section are shown in (a), respectively. Similarly, (b) present the plots of the intersection points in the periodic state of the Duffing system and the Poincaré section, respectively. The red dots represent the intersection points between the Poincaré section and the chaotic oscillator. Evidently, when the Duffing system is in a chaotic state, the values of the Poincaré section demonstrate a high degree of randomness. Conversely, when the Duffing system is in a periodic state, the values of the Poincaré section exhibit minimal fluctuations. By definition, entropy measures the complexity of a system, and the entropy value of the motion trajectory is higher in the chaotic state than in the periodic state. Therefore, the entropy value of the Poincaré section can be used to distinguish between chaotic and periodic states of the system.

### 3.2. Improved Permutation Entropy Algorithm

The Improved Permutation Entropy Algorithm (IPE) improves upon the traditional Permutation Entropy Algorithm (PE) by addressing the issue of missing amplitude information [28]. This algorithm is capable of extracting more information from complex sequences while reducing computational complexity and enhancing signal resolution. The algorithmic flow is as follows:

(1) Normalize the time series $\left\{x_{1},x_{2},\cdots ,x_{N}\right\}$ through the cumulative distribution function shown in the following equation, where μ and $\sigma^{2}$ represent the mean and variance of the time series, respectively.

(4) $$y_{i}=\frac{1}{\sigma \sqrt{2\pi }}\int_{-\infty }^{x_{i}}e^{\frac{-{\left(t-\mu \right)}^{2}}{2\sigma^{2}}}dt$$

(2) Phase space reconstruction.

(5) $$Y_{i}=\left[y_{i},y_{i+\tau },\cdots ,y_{i+\left(m-1\right)\tau }\right]1\leq i\leq N-m+1$$

(3) Symbolize the first column $Y\left(:,1\right)$ of the phase space Y using the Uniform Quantification Operator (UQO) and calculate the corresponding symbolization result for the first column $S\left(:,1\right)$ of the phase space S.

(6) $$UQO\left(u\right)=\left\{\begin{array}{c} 0\\ 1\\ \vdots \\ L-1 \end{array}\right.\begin{array}{c} y_{\min}\leq u\leq \Delta +y_{\min}\\ y_{\min}+\Delta \leq u\leq 2\Delta +y_{\min}\\ \vdots \\ y_{\max}-\Delta \leq u\leq y_{\max} \end{array}$$

Here, *L* denotes a predetermined discretization parameter; Δ represents the discrete interval and meets $\Delta =\left(y_{\max}-y_{\min}\right)/L$; $y_{\max}$ and $y_{\min}$ represent the maximum and minimum values of the sub-sequence y, respectively.

(4) The corresponding symbolization result $S\left(:,k\right)$ for the k column $Y\left(:,k\right)$ (2≤k≤m) of the phase space Y is obtained using the following formula:

(7) $$S\left(j,k\right)=S\left(j,1\right)+floor\left[\left(Y\left(j,k\right)-Y\left(j,1\right)\right)/\Delta \right]1\leq i\leq N-m+1$$

where floor indicates rounding down.

(5) With reference to the symbol patterns’ definition in the Permutation Entropy Algorithm, the improved permutation entropy (IPE) regards every row of the symbolized phase space S as a “pattern” $\pi_{l}$, $1\leq l\leq L^{m}$ and utilizes the term Symbol Pattern (SP) in the algorithm. Calculate the probability $p_{l}$ of each SP in the symbol phase space; according to the definition of Shannon entropy, the improved permutation entropy can finally be expressed as:

(8) $$IPE\left(m,L\right)=-\sum_{l=1}^{L^{m}}p_{l}\ln p_{l}$$

When only one element in the probability distribution of the SP is 1 and the other elements are 0, IPE takes the minimum value 0. When the probability distribution follows a uniform distribution, IPE takes the maximum value $\ln(L^{m})$. Therefore, IPE can be normalized. In this study, normalized entropy values were used.

### 3.3. Threshold Determination Method for Duffing System Based on PMIPE

In this paper, a threshold determination method for the Duffing system based on PMIPE is proposed by combining Poincaré map and IPE. The calculation steps are as follows:

(1) Determine the frequency and other parameters of the Duffing oscillator system based on the signal to be detected by the weak signal detection system.
(2) Impose distinct driving forces on the Duffing oscillator system to induce periodic and chaotic states, respectively.
(3) Calculate the Poincaré section sequences of the Duffing oscillator system, in chaotic and periodic states, correspondingly, to obtain a set of Poincaré section sequences in varied states.
(4) Use the IPE algorithm to calculate the complexity of this set of Poincaré section sequences and obtain the curve of complexity as a function of driving force.
(5) Using entropy = 0.15 as a critical standard, if entropy < 0.15, it is considered that the system is in a stable periodic state, with entropy values exceeding 0.15 defined as non-periodic entropy.
(6) Determine the threshold of the duffing detection system as the maximum driving force that has a non-periodic entropy.

In practical applications, the signal to be detected typically contains noise. As discussed in Section 2.2, the impact of noise on the determination of the threshold can be neglected if the noise is non-stationary or its characteristics are unknown.

**Figure 5:**
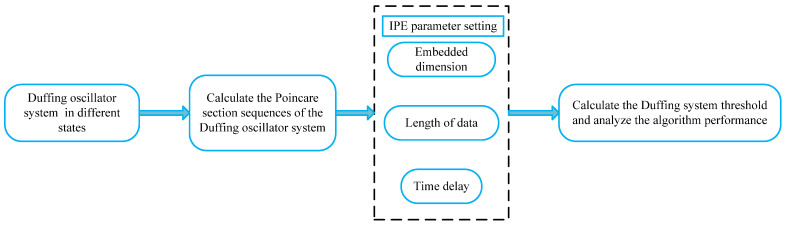
The flowchart of threshold determination method based on PMIPE.

This research approach is illustrated in the flowchart presented in Figure 5.

## 4. Results and Discussion

### 4.1. Influence of Different Parameters on Improved Permutation Entropy

When utilizing the improved permutation entropy to solve the threshold of the Duffing system, it is necessary to select appropriate parameters such as embedding dimension, data length, and time delay so that the IPE of the Poincaré section sequence of the Duffing system can differentiate the sample entropy of chaotic and periodic states.

(1) Influence of Embedding Dimension on IPE

**Figure 6:**
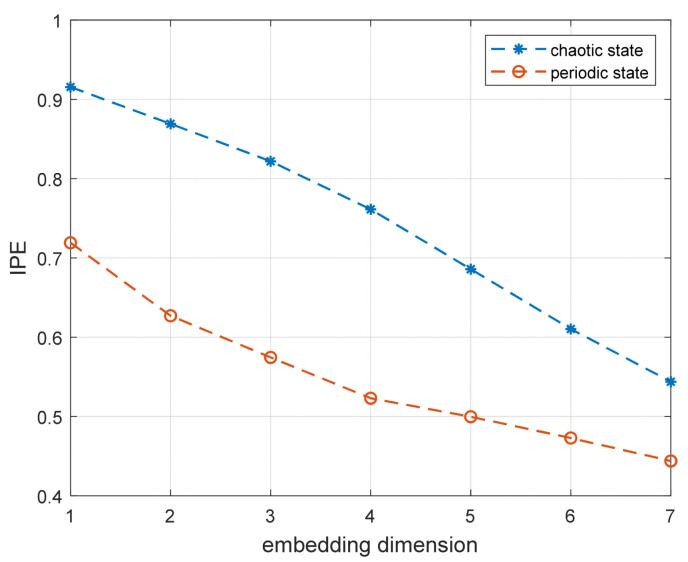
The influence of embedding dimension on IPE algorithm.

To investigate how embedding dimension m influences improved permutation entropy, we set the power frequency of the Duffing system as 10 Hz, sampling interval as 0.001 s, and sampling time as 10 s. Additionally, we set different power values r = 0.838 and r = 0.80, respectively, to put the Duffing system in periodic and chaotic states. We used the IPE algorithm to calculate the Poincaré section sequences of the aforementioned Duffing system, and the results are shown in Figure 6. It can be observed that when the embedding dimension is between 1 and 5, the entropy values exhibit significant disparities. As the embedding dimension increases, the entropy values of both types of Poincaré section sequences diminish, with the entropy value of the chaotic sequence plummeting at a faster rate. Consequently, when computing the Poincaré section entropy value using improved permutation entropy, this investigation suggests selecting an embedding dimension of 1≤m≤5.

(2) Influence of Data Length on IPE

**Figure 7:**
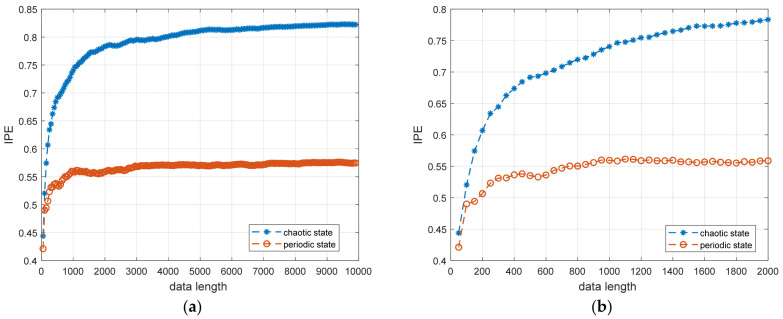
Influence of data length on IPE algorithm. (**a**) Full view; (**b**) partial magnification view.

We set the Duffing system as in Step (1) in this Section and calculated the entropy values of the periodic and chaotic states for 100 to 10,000 Poincaré section points. Figure 7a depicts the influence of diverse data lengths on the IPE algorithm, while Figure 7b offers a local zoom-in. Notably, for data lengths above 500, the entropy values for periodic and chaotic states attain a stable tendency. Such tendencies facilitate differentiating system states based on entropy values.

(3) Influence of time delay on IPE

**Figure 8:**
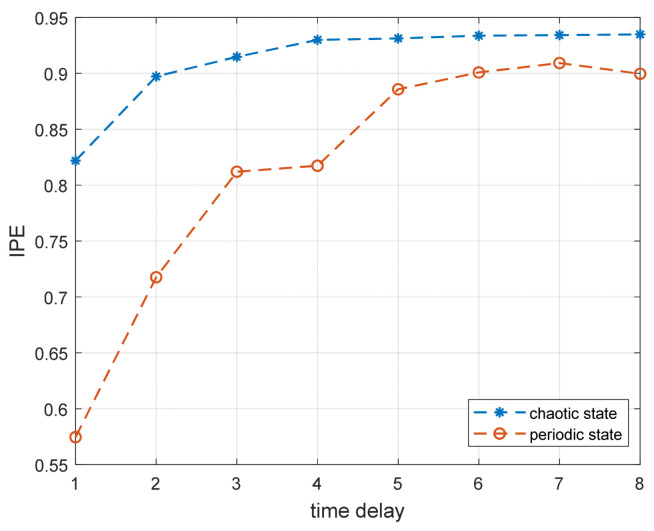
The influence of time delay on IPE algorithm.

We set the Duffing system as in Step (1) in this Section and calculated the entropy values of the periodic and chaotic states for time delays ranging from 1 to 8 points. Figure 8 depicts the impact of time delay on the IPE algorithm. When the time delay is between 1 and 4 points, the entropy values have significant differences. As the time delay increases, the entropy value of the Poincaré section sequence of the chaotic system also rises, tending toward stability. Comparatively, the entropy value of the periodic sequence experiences faster growth. Notably, prolonged time delays lead to a loss of the periodic sequence’s characteristic information. Therefore, this study recommends selecting a small time delay, preferably not exceeding 4 points, when calculating Poincaré section entropy values using improved permutation entropy.

### 4.2. Simulation of Threshold Determination for Duffing Oscillator System with Different Frequency and Driving Forces

**Figure 9:**
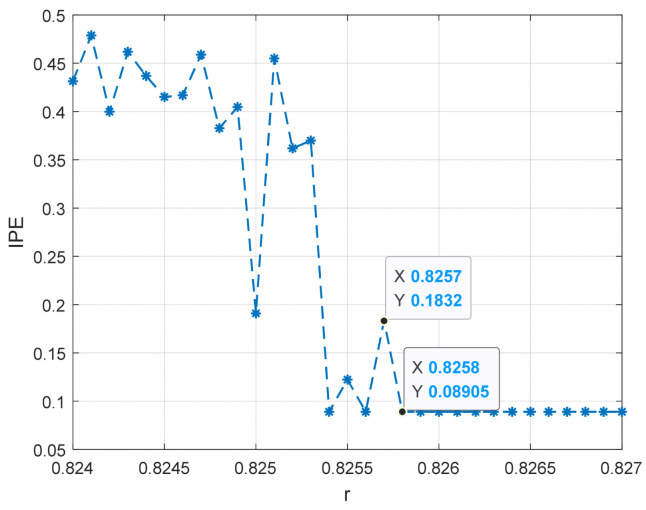
The entropy change of the 10 Hz signal detection system.

**Figure 10:**
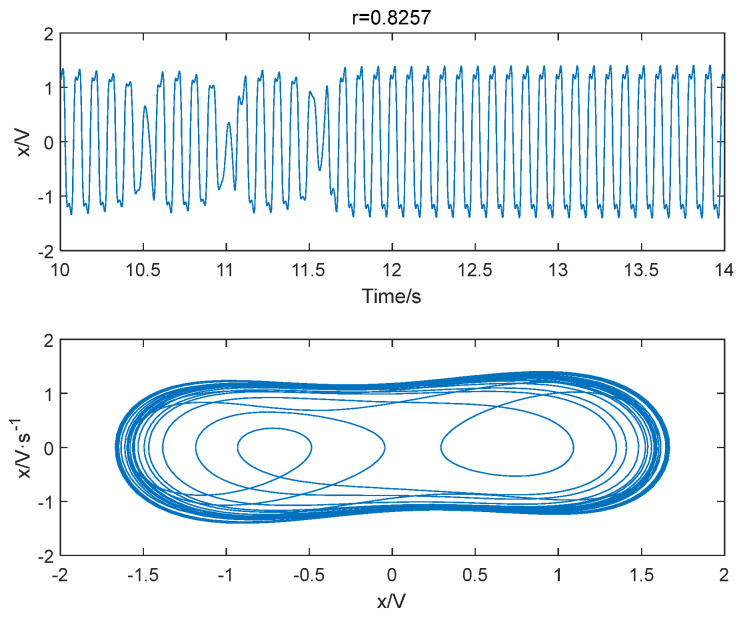
When f = 10 Hz and r = 0.8257, the system is in a chaotic state.

**Figure 11:**
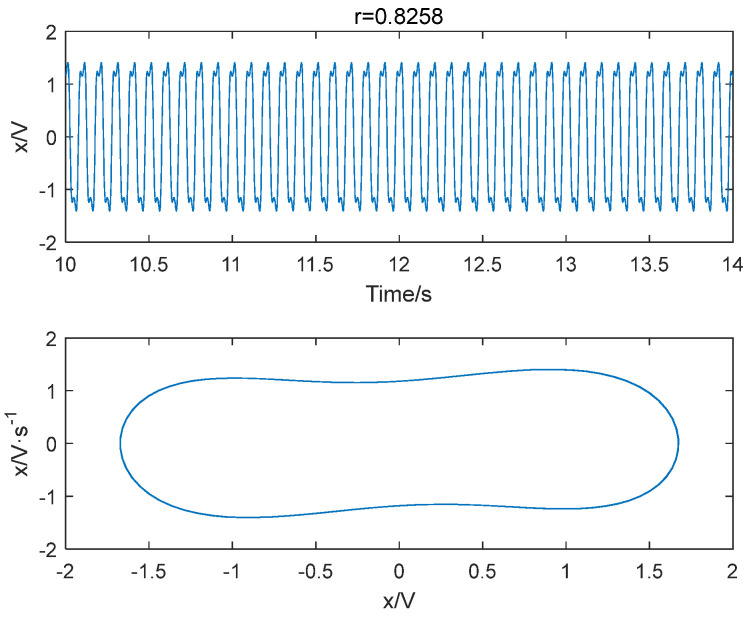
When f = 10 Hz and r = 0.8258, the system is in a periodic state.

Sinusoidal driving forces with varying frequencies and amplitudes were applied to the system with a sampling time ranging from 10 to 30 s and a sampling interval of 0.001 s. The system was subjected to driving forces with a range from 0.824 to 0.827 with an interval of 0.001. Notably, we also consider a larger range of r, please see Appendix A for detailed results. Based on the conclusion in Section 4.1, the embedding dimension m was set to 4 and the time delay set to 1 point, and the data length was greater than 500. The improved permutation entropy of the system is analyzed with respect to the amplitude of a 10 Hz sinusoidal signal, as shown in Figure 9. The analysis shows that the improved permutation entropy decreases and then stabilizes after the driving force amplitude exceeds 0.8257. The system state when r = 0.8257 is shown in Figure 10. The critical threshold of the system is determined to be r = 0.8257 using the IPE-Poincaré method. Figure 11 shows that the system is in a periodic state when r = 0.8258, which proves that the threshold should be 0.8257.

**Figure 12:**
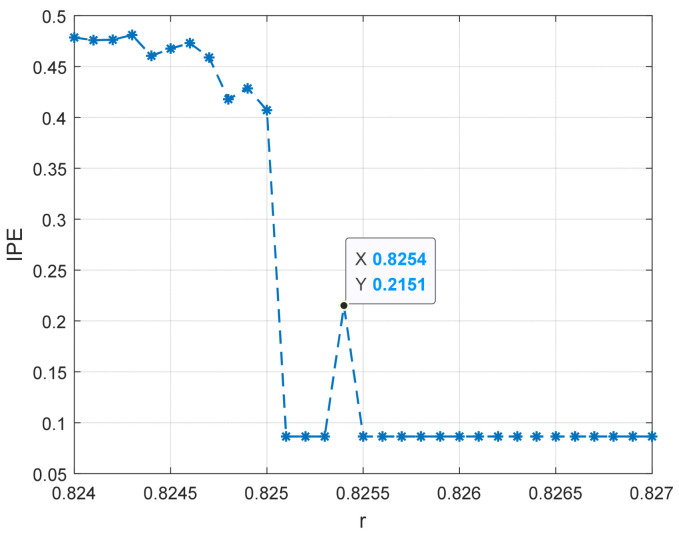
The entropy change of the 20 Hz signal detection system.

**Figure 13:**
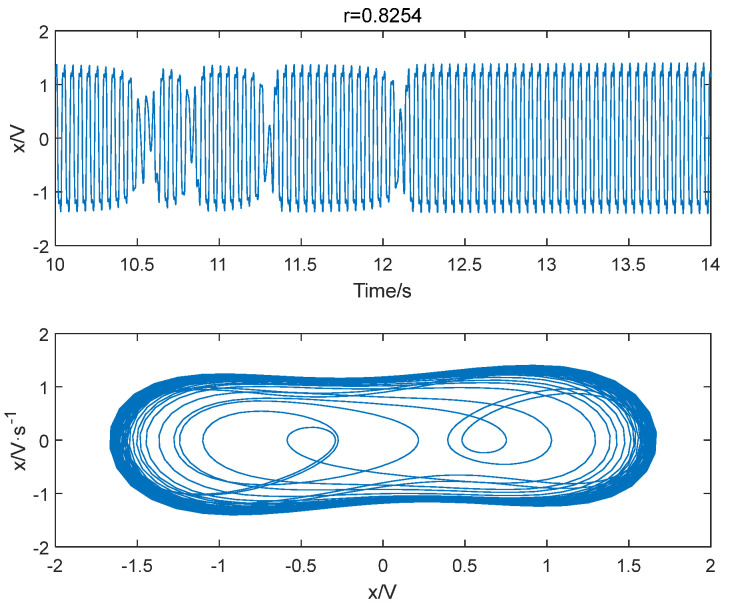
When f = 20 Hz and r = 0.8254, the system is in a chaotic state.

**Figure 14:**
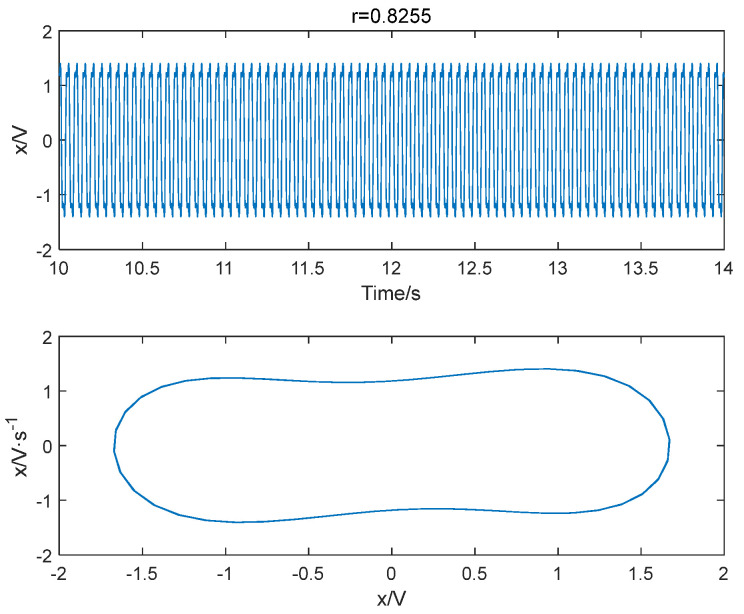
When f = 20 Hz and r = 0.8255, the system is in a periodic state.

The improved permutation entropy of a 20 Hz sinusoidal signal detection system is analyzed with respect to the driving force amplitude, as shown in Figure 12. The analysis indicates that the improved permutation entropy decreases and then stabilizes after the driving force amplitude exceeds 0.8254. The system state when r = 0.8254 is shown in Figure 13, where a chaotic state can be easily found. Hence, the critical threshold of the system is determined to be r = 0.8254 using the IPE-Poincaré method. Figure 14 shows that the system is in a periodic state when r = 0.8255, which proves that the threshold should be 0.8255.

**Figure 15:**
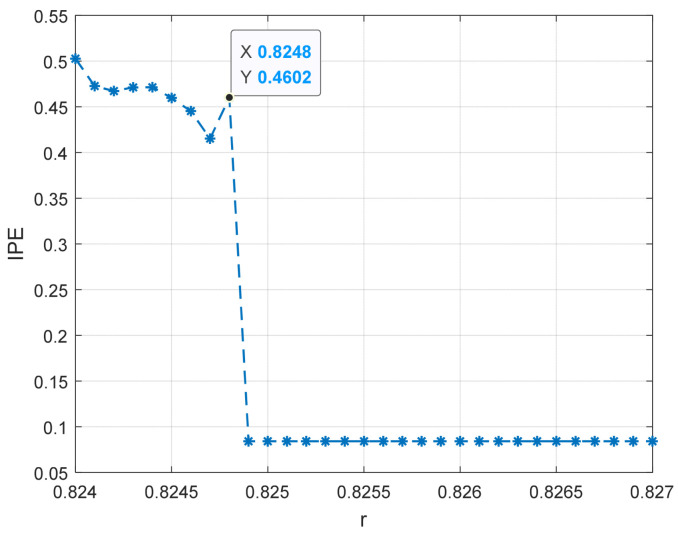
The entropy change of the 100 Hz signal detection system.

**Figure 16:**
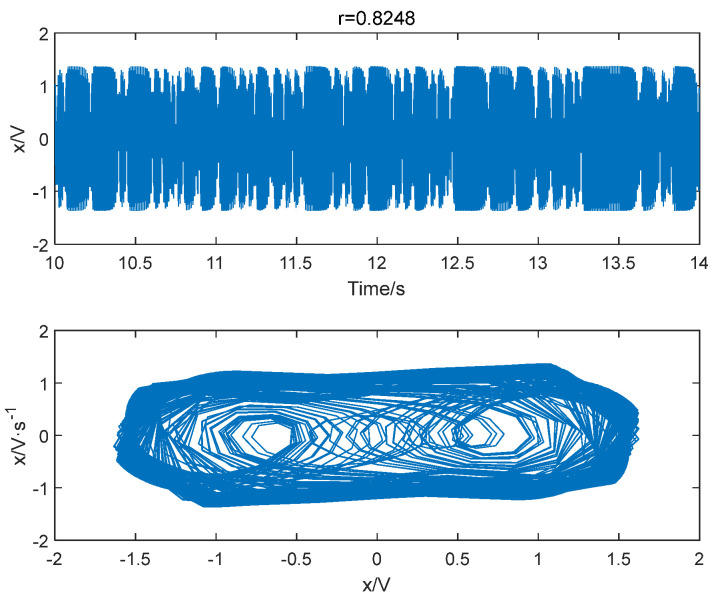
When f = 100 Hz and r = 0.8248, the system is in a chaotic state.

**Figure 17:**
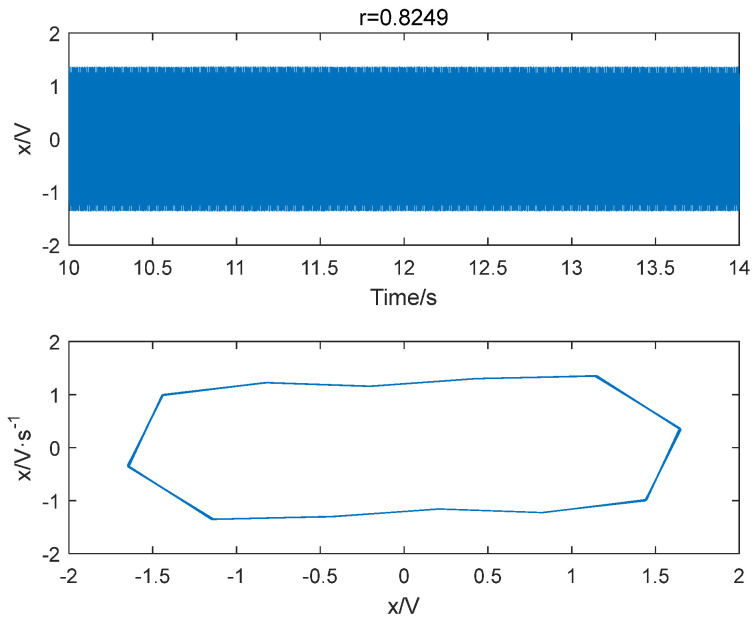
When f = 100 Hz and r = 0.8249, the system is in a periodic state.

Similarly, the improved permutation entropy of a 100 Hz sinusoidal signal detection system is analyzed with respect to the driving force amplitude, as shown in Figure 15. The analysis shows that the improved permutation entropy decreases and then stabilizes after the driving force amplitude exceeds 0.8248. The system state when r= 0.8248 is shown in Figure 16. Therefore, the critical threshold of the system is determined to be r= 0.8248 using the IPE-Poincaré method. Figure 17 shows that the system is in a periodic state when r= 0.8249, which proves that the threshold should be 0.8248. Table 2 compares the true thresholds with the results obtained from Figure 9, Figure 10, Figure 11, Figure 12, Figure 13, Figure 14, Figure 15, Figure 16 and Figure 17; it can be seen that, for all three cases mentioned above, our method provides a very accurate evaluation.

### 4.3. Comparison and Analysis of Different Methods

**Figure 18:**
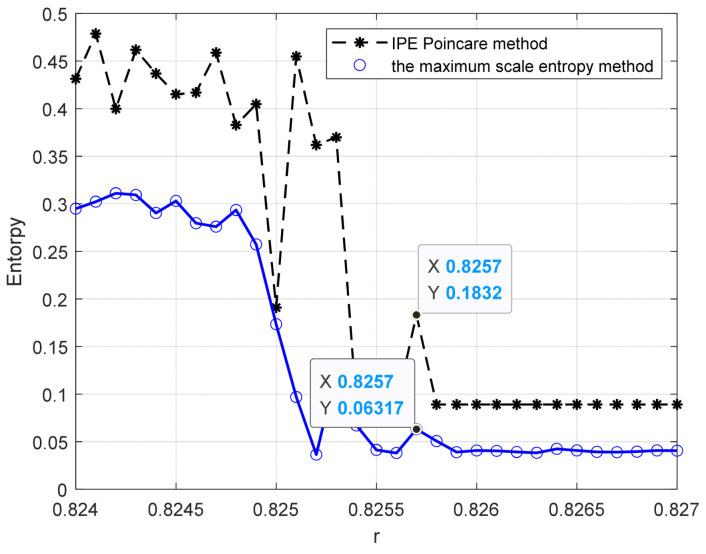
Results of threshold calculation by different methods.

**Figure 19:**
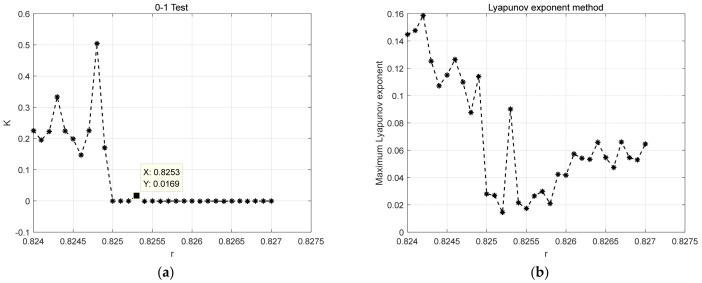
Results of threshold calculation by 0–1 test and Lyapunov exponents. (**a**) 0–1 test; (**b**) Lyapunov exponents.

For the 10 Hz sinusoidal signal detection system, the maximum multi-scale entropy method from literature [19] and the PMIPE method proposed in this paper were used to calculate the system threshold, with a Duffing sequence length of 400,000. For the maximum multi-scale entropy method, the length of each sub-sequence was set to 30,000, the initial number of chromosomes to six, and the crossover point to a random number. The calculation outcomes are presented in Figure 18. It is noteworthy that both methods can correctly determine the critical threshold of the system. However, the maximum multi-scale entropy method required 573.95 s to calculate, while the IPE-Poincaré method took only 30.69 s. Thus, the IPE-Poincaré method significantly reduces the computational complexity, rendering it more suitable for real-time computations. For comparison, we also use the 0–1 test method and Lyapunov exponents to evaluate the threshold; results are given in Figure 19. Obviously, the 0–1 test value becomes constant after r = 0.8253, meaning that its threshold evaluation result is 0.8253, which is not very accurate. As for the Lyapunov exponent method, it assigns higher values to chaotic states and smaller values to periodic states. However, it is hard to determine an accurate threshold. This may because of the inappropriate parameter selection; therefore, the calculation of Lyapunov exponents can be easily influenced by parameter selection. Table 3 compares the results of the abovementioned four methods. It can be seen that the proposed method and MSE achieve the highest evaluation accuracy, followed by the 0–1 test and Lyapunov exponent. Notice that our method only takes 30.69 s to complete this experiment while all of the other methods require more than 500 s. Hence, our method outperforms traditional algorithms in both accuracy and computation cost.

### 4.4. Verification of the Real Underwater Acoustic Signal

**Figure 20:**
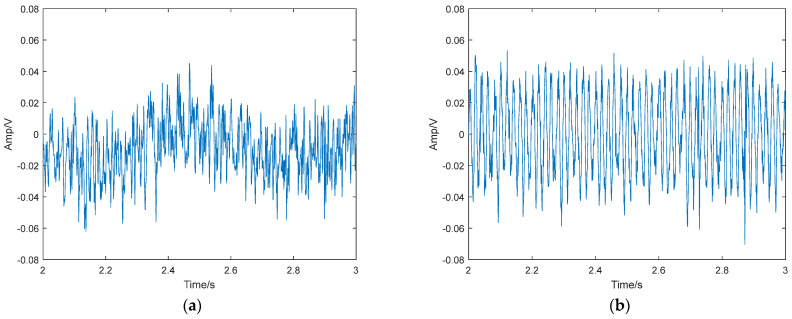
Real underwater acoustic signal. (**a**) Ship signals; (**b**) ambient noise.

**Figure 21:**
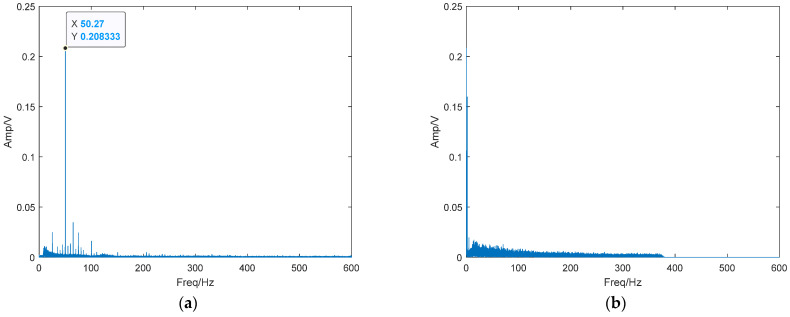
Spectrum of real underwater acoustic signals. (**a**) Ship signals; (**b**) ambient noise.

**Figure 22:**
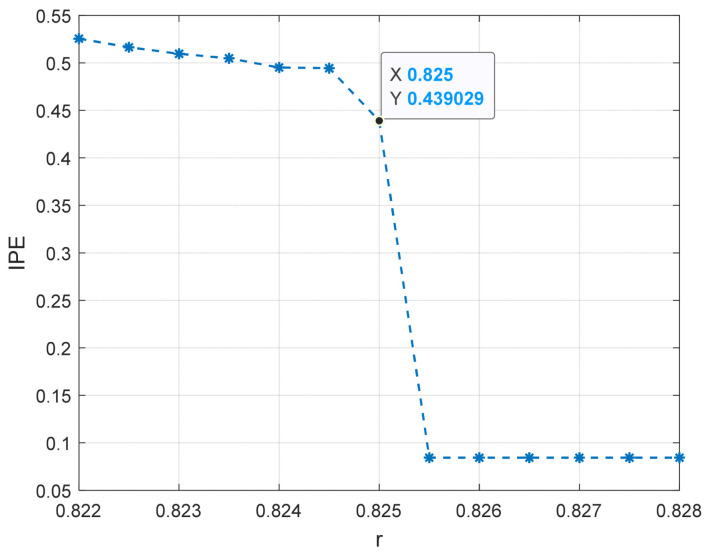
The entropy change of the real signal detection system.

To verify the effectiveness of the threshold determination method based on PMIPE in solving real underwater acoustic detection systems, a set of measured ship signals and ambient noise was selected as sample data. The waveform of the measured data is shown in Figure 20, and its frequency domain waveform is shown in Figure 21. It can be seen that the measured underwater acoustic ship signal contains a sinusoidal signal with a frequency of 50.27 Hz. Using the same method as in this paper, we set the frequency of 50.27 Hz in the duffing oscillator system. The system threshold can be obtained as r = 0.825, as shown in Figure 22.

**Figure 23:**
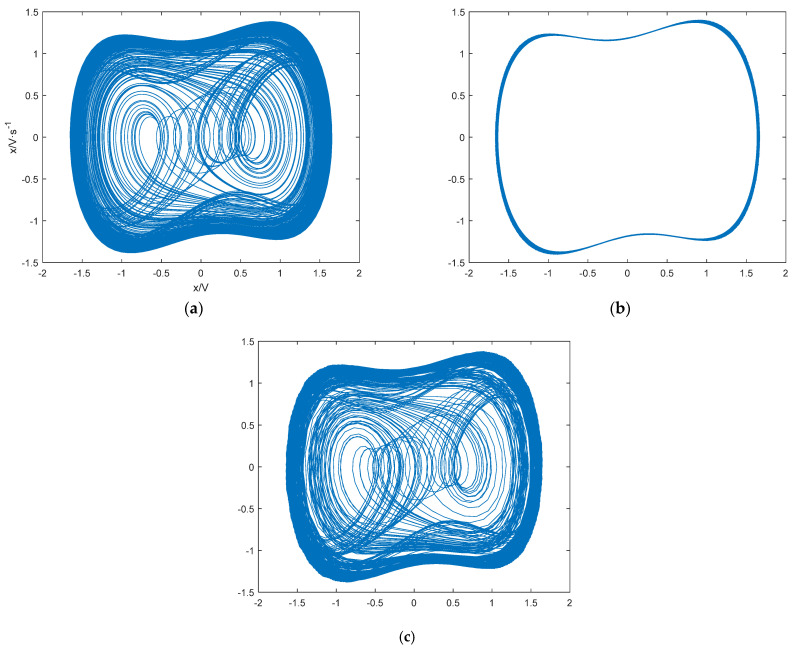
Detecting real underwater acoustic signals. (**a**) The duffing system did not add a real underwater acoustic signal; (**b**) the duffing system adds the ship signals; (**c**) the duffing system adds ambient noise.

We adjusted the driving force amplitude r to 0.825 so that the system was in a critical chaotic state, and then we added the real underwater acoustic signal to the detection system. When we added the ship signals to the system, the system phase diagram transitioned from the chaotic state shown in Figure 23a to the periodic state shown in Figure 23b. When we added the ambient noise to the system, the system phase diagram transitioned from the chaotic state shown in Figure 23a to the periodic state shown in Figure 23c. Comparing Figure 23b,c shows that the system realized the detection of target signals among real underwater acoustic signals. Therefore, the PMIPE method can accurately calculate the system threshold.

### 4.5. Analysis of Anti-Noise Performance of Threshold Determination Methods

**Figure 24:**
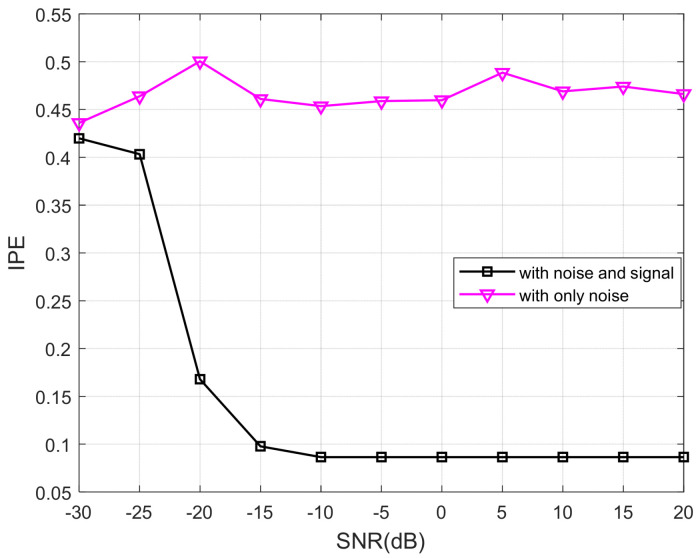
IPE results of chaotic state and periodic state under different SNRs.

For the 20 Hz sine signal Duffing oscillator detection system, with the driving force amplitude set to r = 0.8254, a same-frequency signal with an amplitude of 0.001 V was added to the system as the detected signal, which put the system in a periodic state. We then added noise signals with different signal-to-noise ratios (SNRs) to the detected signal and calculated the IPE-Poincaré value to assess the change in IPE under different SNRs in both chaotic and periodic states. The results are shown in Figure 24. It can be observed that when the SNR is greater than 20 dB, noise has little influence on the IPE-Poincaré value. As the SNR decreases, the critical threshold for chaos increases due to the amplification of external noise. Despite this, the system remains in a chaotic state due to the small amplitude of the driving force signal. It has a similar entropy value to that of a system with only noise signals but no same-frequency signals. Therefore, when the SNR is greater than −20 dB, the periodic state of the system can be effectively determined, achieving the goal of signal detection.

## 5. Conclusions

The problem addressed in this paper revolves around the challenging task of determining the threshold in the Duffing system. Emphasis is placed on leveraging the substantial distinctions between the Poincaré section values in both periodic and chaotic states of the Duffing system. An advanced permutation entropy of the Poincaré section composition sequence is employed to establish a correlation between the driving force amplitude and the improved permutation entropy. The utilization of a PMIPE-based threshold determination technique is then introduced, and this algorithm is applied to the detection system of sinusoidal signals with different frequencies. A comparative analysis is conducted with the maximum multiscale entropy, 0–1 test, and Lyapunov exponent methods to gauge the efficacy of the proposed technique. The results of our investigation illustrate that the algorithm we propose exhibits a remarkable accuracy to determine the system’s threshold. Moreover, our method needs much less computational cost compared with the traditional methods, and our algorithm also performs well under noisy conditions. In summary, our study provides an innovative and effective resolution to the intricate challenge of threshold determination in the Duffing system, showcasing the potential of the proposed algorithm in addressing this pertinent issue.

## Figures and Tables

**Table 1:** Weakness of traditional threshold determination methods.

Methods	Weakness
Melnikov analysis	High implementation difficulty
Phase diagram method	Poor computational accuracy
Power spectrum method	Unable to distinguish between periodic and chaotic dynamics
Poincaré section method	Subjective
0–1 test	Poor computational accuracy

**Table 2:** Comparison of true thresholds with the results obtained by our method.

Frequency	True Threshold	Evaluated Threshold by Our Method
10 Hz	0.8257	0.8257
20 Hz	0.8254	0.8254
100 Hz	0.8248	0.8248

**Table 3:** Comparison of traditional threshold determination methods with our method.

Methods	Frequency	True Threshold	Evaluated Threshold	Computation Time (s)
MSE	10 Hz	0.8257	0.8257	573.95
0–1 test	10 Hz	0.8257	0.8253	598.38
Lyapunov	10 Hz	0.8257	\	561.57
Our method	10 Hz	0.8257	0.8257	30.69

**Figure A1:**
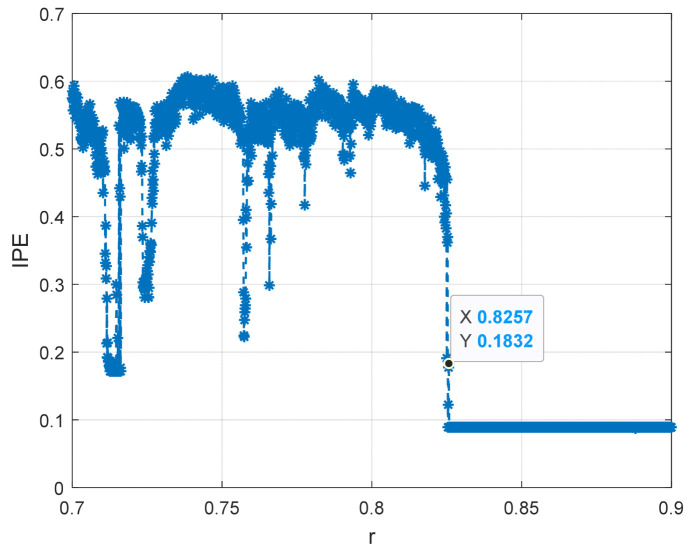
The entropy change of the 10 Hz signal detection system. The driving force ranges from 0.7 to 0.9, with a step length of 0.001.

**Figure A2:**
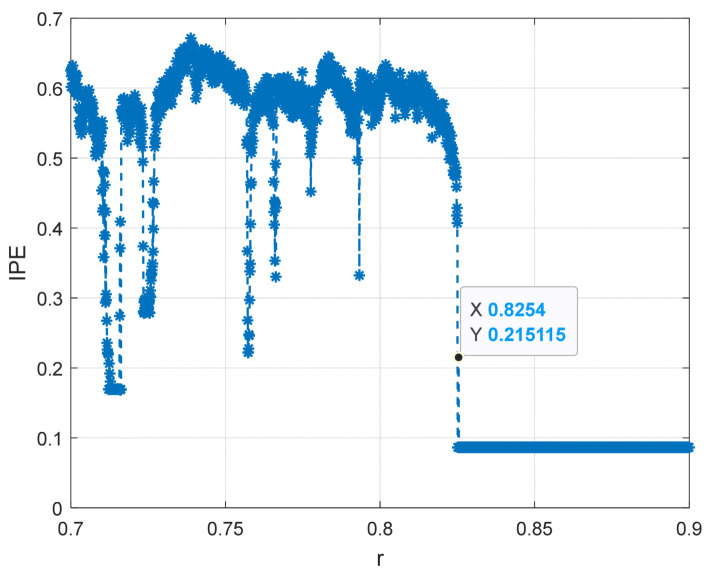
The entropy change of the 20 Hz signal detection system. The driving force ranges from 0.7 to 0.9, with a step length of 0.001.

**Figure A3:**
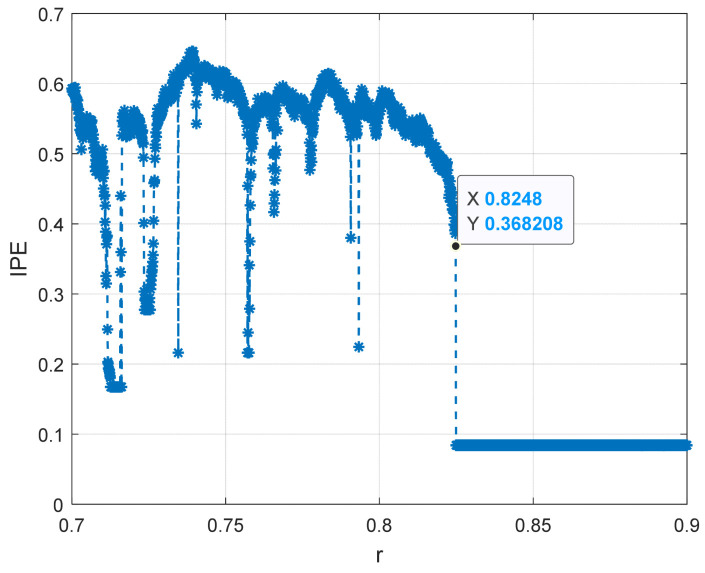
The entropy change of the 100 Hz signal detection system. The driving force ranges from 0.7 to 0.9, with a step length of 0.001.

**Figure A4:**
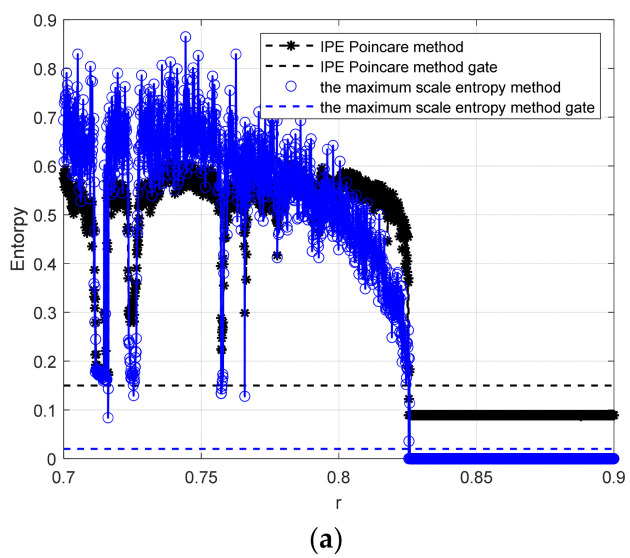

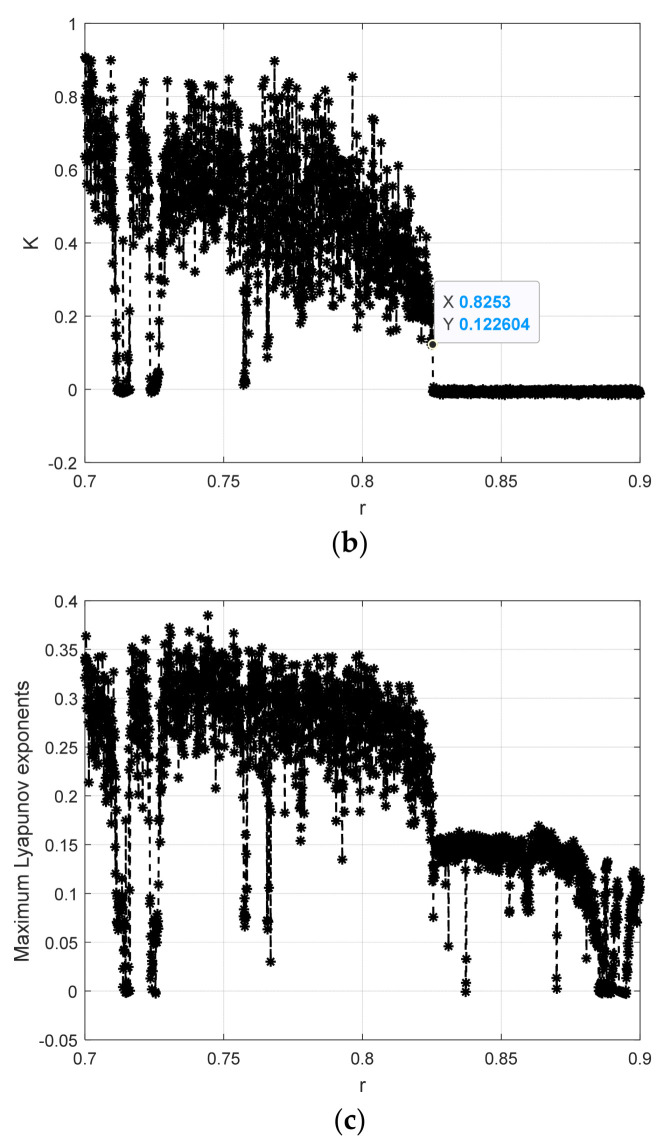
Threshold evaluation results by different methods for signal detection system operating at 10 Hz: (**a**) results of MSE and our algorithm; (**b**) results of 0–1 test; (**c**) results of Lyapunov exponent.

## Data Availability

The datasets analyzed during the current study are available from the corresponding authors on reasonable request.

## Appendix A

In this section, we extend the range of the driving force amplitude. A comprehensive analysis of the system output is undertaken for each distinct driving force amplitude condition.

Figure A1, Figure A2 and Figure A3 show how the IPE varies across a broader range of the driving force for signal detection systems operating at frequencies of 10 Hz, 20 Hz, and 100 Hz, respectively. The step-length is set as 0.001. For all three cases, our method consistently yields higher IPE values when the driving force is less than 0.82, indicating a heightened complexity in the dynamics within this range. As the value of r increases, a notable abrupt decline in IPE occurs, signifying a transition of the system from a critical chaotic state to a periodic state. Subsequently, the IPE stabilizes at a lower constant level. According to our threshold determination strategy, the recommended thresholds for the signal detection systems operating at frequencies of 10 Hz, 20 Hz, and 100 Hz are determined to be 0.8257, 0.8254, and 0.8248, respectively. Notably, these values align seamlessly with the results expounded upon in Section 4.2 of this paper.

Figure A4 compares the results of threshold evaluations from various methods applied to a signal detection system operating at 10 Hz. Once again, these findings corroborate those detailed in Section 4.3 of this paper. Notably, both MSE and our algorithm converge on a threshold value of 0.8257, while the 0–1 test yields a slightly different assessment at 0.8253. The application of the Lyapunov exponent method proves challenging in threshold determination, possibly attributed to the sensitivity of the results to parameter selection.
